# Psychological distress is influenced by length of stay in resettled Iraqi refugees in Australia

**DOI:** 10.1186/s13033-016-0036-z

**Published:** 2016-01-20

**Authors:** Maria Gabriela Uribe Guajardo, Shameran Slewa-Younan, Mitchell Smith, Sandy Eagar, Glenn Stone

**Affiliations:** Mental Health, School of Medicine, University of Western Sydney, Sydney, Australia; New South Wales Refugee Health Service, Liverpool, Australia; Centre for Research in Mathematics, School of Computing, Engineering and Mathematics, University of Western Sydney, Sydney, Australia

**Keywords:** Iraqi refugees, Psychological distress, Length of stay, Australia

## Abstract

**Background:**

Psychological distress has been well identified in recently resettled refugee groups; however, evidence on psychological distress over time is not conclusive. Australia has welcomed a large refugee population in recent decades, including Iraqis who currently form one of the largest groups being resettled in Australia.

**Methods:**

This study aimed to explore psychological distress in two samples of Iraqi refugees, those who recently arrived (n = 225, average length of stay = 0.55 months) and those with a longer period of resettlement (n = 225, average length of stay = 58.5 months). To assess general symptoms of anxiety and depression, the Kessler Psychological Distress Scale was employed. Associations between participants’ demographic characteristics and psychological distress levels were examined.

**Results:**

A significant difference between groups, *t* (441) = −2.149, p = 0.0324, was found, indicating that study participants with longer periods of resettlement were experiencing higher levels of psychological distress than recent arrivals.

**Conclusion:**

Our findings have implications for both for government and non-government funded organisations who should consider the provision of assistance programs beyond the initial arrival period.

## Background

A major refugee crisis is taking place in the Middle East with more than 5 million individuals categorised as a population of concern to the United Nation Higher Commission for Refugees (UNHCR) in this region alone [1]. A global estimation report indicated that at the end of 2013, there were 51.2 million people forcibly displaced by persecution and conflict [2]. A refugee is a ‘person who has left their home country as a result of a well-founded fear of persecution for reasons of race, religion, nationality, membership of a particular social group or political opinion’ (p. 5) [3]. Refugee populations have commonly experienced torture, war or civil unrest, the loss of family and friends through violence, prolonged periods of deprivation and migration trajectory difficulties [3]. Iraq and its population has been reported as one of the most affected nations worldwide, plagued by a long period of violence, repression and human rights violations including torture [4]. Introduced in the early 1980s, the Political Terror Scale (PTS) is a measure of state terror and political violence of basic human rights based on data collected by Amnesty International and the US State Department [5]. PTS provides an annual assessment using a point scale, with scores of 4 or higher representing countries in which murders, disappearances and torture are common and affect the whole population [5]. Iraq scored 5 on the PTS scale in 2014, making this nation one of the top five worst offender countries for human security [5].

Since World War II, Australia has welcomed over 750,000 people from many different countries in response to changing global resettlement and humanitarian needs [6]. Australia has a humanitarian program for refugees and other humanitarian entrants that aims to provide permanent resettlement to individuals who have been forced to leave their homes by armed conflict, persecution and human rights abuses [7]. It is important to note that there are two main components of Australia’s Humanitarian Program, the offshore and onshore. Offshore applications offer resettlement to refugees and humanitarian entrants from overseas under two categories. Most offshore refugees are referred to Australia by the UNHCR and are formally accepted and resettled under the ‘Refugee’ category. These entrants have been assessed and accepted as refugees under ‘Refugee Convention’ criteria. The Special Humanitarian Program (SHP) also offers resettlement to those located offshore, who, while not necessarily being refugees, face human rights abuses in their home country and have a connection with Australia. Applicants must have a sponsor (e.g. a permanent resident, Australian citizen or organisation). The onshore component offers protection to people who have arrived in Australia, lodged an asylum claim, and subject to being found to meet the criteria as refugees under the Refugee Convention are granted protection [7]. While awaiting the processing of onshore humanitarian claims, these persons are known as asylum seekers. In this study all participants included were those offered permanent protection in Australia, with asylum seekers excluded. Over the period 2003–2004 to 2012–2013 Australia resettled 146,321 refugees [8]. Most recently, the Australian Humanitarian Program 2013–2014 granted 13,768 visas to onshore and offshore visa applicants [9]. Currently, Iraq is one of the top source countries of refugee applications to Australia [9].

General psychological distress and, in particular, posttraumatic stress disorder (PTSD) and major depressive episode (MDE) have been long established as common mental health concerns amongst refugee populations [10, 11]. In one of the largest meta-analyses to date of mental health among resettled refugees, Steel and colleagues noted that weighted prevalence rates across the studies were 30.6 and 30.8 % for PTSD and depression, respectively [11]. A recent systematic review on refugees of Iraqi background resettled in Western countries reported that PTSD rates vary from 8 to 37.2 % and depression rates from 28.3 to 75 % [12]. Comparatively, these rates well exceed the 12-month prevalence rates for PTSD and depression of 6.4 and 4.1 %, respectively, in the general Australian population [13].

An important area that needs to be addressed when developing resettlement programs for refugees is the stability or change in psychological distress levels over time. To date, research literature has demonstrated contradictory evidence. Some researchers have supported the idea of positive outcomes in the mental health of resettled refugee populations over time. Specifically, Porter and Haslam (2005) indicated that a greater time between date of study and time of displacement was associated with better mental health for refugees compared to the control group in their meta-analysis [14]. In line with these findings, Steel and colleagues noted that trauma-related mental illness seemed to reduce steadily over time in Vietnamese refugees resettled in Australia [15]. In addition, Krupinski, Stoller and Wallace (1973) found that some Jewish refugees resettled in Australia had lower rates of mental health disorders, specifically schizophrenia, compared to other Eastern European refugee groups, despite their high level of exposure to traumatic events [16]. Further, there is evidence of improved mental health outcomes over time when interventions were implemented [17–19]. In contrast, other studies have indicated greater psychological distress over time. Kyrmayer and colleagues (2011) noted that although the prevalence of mental health disorders was initially lower, this increased over time [20]. A cross-sectional study conducted with Cambodian refugees resettled in the US reported prevalence rates of mental health disorders 20 years after resettlement, with 62 % for PTSD and 51 % for depression [21]. This would suggest that trauma related disorders remain problematic many years post resettlement in some refugee groups. Hence it may be concluded that psychological distress more generally and trauma-related disorders specifically, may persist for many years and in some cases even increase over time [21].

Consequently, this study sought to contribute to this literature by exploring levels of psychological distress over time in two samples of Iraqi refugees with differing lengths of resettlement in Australia. Given the mixed direction of evidence on psychological distress levels over time in resettled refugees, there were no specific directional predictions. Instead, we postulated that some differences in psychological distress levels would be noted between the two samples of Iraqi refugees.

## Methods

### Study design and participants

This study had a total sample of 450 Iraqi refugee adults, all granted permanent residence in Australia.

Group one consisted of 225 Iraqi refugee adults (mean age = 38, SD = 13.28) with relatively short lengths of stay in Australia. This sample was recruited through a review of medical records from the New South Wales Refugee Health Service (NSW RHS). These data were routinely collected by NSW RHS based as part of a program that aims to screen all new refugees arriving in Sydney for mental and physical health conditions commonly experienced by those of refugee background. Assessments were conducted from November 2012 to July 2013 by nurses from the NSW RHS Refugee Nurse Program, using accredited professional interpreters whenever required.

Group two included 225 Iraqi refugee adults (mean age = 38, SD = 14.02) with longer lengths of stay, residing in Sydney who were attending the Adult Migrant English Program (AMEP) in Western Sydney. This group was part of a larger study that sought to examine the mental health literacy and psychological status of Iraqi refugees [22]. Participation entailed the completion of a pen-and-paper, respondent-based survey, conducted on the college campuses by means of in-person interviews. Data collection was conducted by two bilingual (fluent in Arabic and English) members of the mental health literacy research team.

Ethics approval for the current study was granted by the South Western Sydney Local Health District Human Research Ethics committee (LNR/14/LPOOL/319; LNRSSA/14/LPOOL/320).

### Measures

#### General psychological distress

To assess general symptoms of anxiety and depression, the Kessler Psychological Distress Scale (K10) [23] was employed. The K10 is a self-report questionnaire of depression and general mental disorder. Scores range from 10 to 50, with established thresholds of low to mild (10–21), moderate (22–29) and severe distress (≥30) applied to provide a measure of symptoms among the participants. The K10 is available in Arabic and has good psychometric properties with internal consistency of α = 0.86 reported for Arabic speaking refugees [24]. Cronbach’s alpha in the current study was 0.941.

#### Demographic profile

Limited demographic data such as marital status, age, gender and time since resettlement were also collected and analysed.

### Statistical analyses

Comparisons between the two groups were made using the independent sample *t* test, two-way ANOVA and t tests with a single step *p* value adjustment for multiple testing [25], as appropriate. Statistical analysis was carried out using R Environment for Statistical Computing 3.1.1. [26]. Continuous variables are presented as means, whereas categorical variables are expressed as percentage (%) frequencies for groups one and two. Missing data were low at 1.1 % and were handled by listwise deletion. Where possible, data on levels of general psychological distress in the wider general Australian community derived from the Australian Health Survey (AHS) 2011–12 [27] were used for comparative purposes. Specifically, Pearson *χ*^*2*^ was performed, comparing the distribution of psychological distress categories in the study participants to reconstructed AHS counts, with the assumption that there was a sampling error, albeit small that is consistent with the approximate sample size reported for the AHS.

## Results

Demographic data from the participants is presented in Table 1.

**Table 1:** Demographic and psychological profile

	Iraqi refugees			
Characteristics	Shorter length stay N^a^	%	Longer length stay N^a^	%
Gender				
Male	108	48.2	93	42.4
Female	116	51.8	126	57.6
Age in years, mean (SD)	38 (SD = 13.3)	–	38 (SD = 14)	–
Months in Australia, mean (SD)	0.55 (SD = 0.7)	–	58.5 (SD = 64.6)	–
Marital status				
Non indicated	22	9.8	6	2.7
Never married	59	26.3	51	23.3
Married	127	56.7	145	66.2
Divorced	3	1.3	5	2.2
Widowed	13	5.8	12	5.5
K10 psychological distress				
Mean (SD)	23.69 (SD = 11.3)	–	25.95 (SD = 10.9)	–
Low to mild	105	47.1	87	39.7
Moderate	48	21.5	45	20.6
Severe	70	31.4	87	39.7

^a^Does not equal to 250 due to missing data

### Differences between groups

Levels of psychological distress reported were significantly different between the two groups [*t* (441) = −2.149*, p* = 0.0324] with higher levels of psychological distress in those Iraqi refugees that have been in Australia for longer.

### Association between participants’ demographic characteristics and psychological distress

A significant group-by-age range interaction (F (5431) = 2.80, p < 0.01649) was found for psychological distress levels. Follow up t tests indicated those aged 45–54 in the longer length of stay group had significantly higher average levels of psychological distress (*p* = *0.00273*). No other interactions between demographic characteristics and psychological distress levels were found to be significant.

Table 2 presents the levels of psychological distress in both groups by demographic subcategories.

**Table 2:** Psychological distress levels by demographic categories

	Iraqi refugees			
Characteristics	Shorter length stay N^a^	Mean K10 Score (SD)	Longer length stay N^a^	Mean K10 Score (SD)
Age (years)				
18–24	50	20.2 (8.2)	49	25.3 (11.7)
25–34	48	19.6 (10.4)	56	20.4 (7.9)
35–44	50	27.7 (12.9)	37	27.5 (11.1)
45–54	45	25.2 (11.1)	43	33.0 (8.4)
55–64	26	28.4 (10.6)	26	25 (11.8)
65–74	5	18.8 (7.1)	8	25.3 (10.8)
Marital status				
Never married	59	21.3 (9.8)	51	23.9 (11.2)
Married	127	25.2 (11.6)	145	26.0 (10.7)
Divorced	3	23.6 (12.1)	5	30.6 (13.0)
Widowed	13	28.6 (13.6)	12	31.9 (10.0)
Gender				
Male	108	22.6 (11.3)	93	25.1 (10.8)
Female	116	24.6 (11)	126	26.5 (10.9)

^a^Does not equal 250 due to missing data

### Psychological distress reported on Australian population and Iraqi refugees

A highly significant difference in psychological distress was noted when comparing the proportions of Iraqi refugees (both groups combined) to the general Australian population using data from the AHS 2011–12 [27] on the K10 categories (*χ*^*2*^*(2, N* = *10,000) 966, p* < *0.0001).* Specifically, there were a greater proportion of Iraqis demonstrating severe levels of psychological distress as reflected in a K10 score of greater or equal to 30, than the general Australian sample (*χ*^*2*^*(1, N* = *10,000) 786.5, p* < *0.0001).* Interestingly, the converse was noted in the low to mild distress category of the K10, where a significantly more proportion of the general Australian sample scored compared to the Iraqi refugees (*χ*^*2*^*(1, N* = *10,000) 804.5, p* *<* *0.0001).*

## Discussion

Resettled Iraqi refugees have been identified as a highly vulnerable group with reports indicating they present with higher degrees of emotional trauma and poorer health compare to other refugee groups [28]. However, evidence on psychological distress levels over time in refugees has been unclear. While some studies proposed a positive association between longer length of resettlement and good mental health [14–16], others argued that length of stay in a host country can negatively impact mental health in refugees with chronic or increased levels of psychological distress over time [20, 21].

This study sought to further explore this area by comparing levels of psychological distress reported in two samples of Iraqi refugees in Australia for differing lengths of time. Additionally, we described the impact of certain demographics factors on levels of psychological distress in the two groups of refugees. Finally, level of psychological distress in the overall refugee group was compared with that in the general Australian population. Our results demonstrated that those refugees with the longer period of resettlement in Australia were more distressed than those with a shorter length of stay. In particular the observed difference was greater in the 45–54 age group. Other variables such as gender or marital status did not statistically influence levels of psychological distress in either group. Finally, levels of severe psychological distress were significantly higher among Iraqi refugees compared to the general Australian population.

This study supports the notion that the period following initial resettlement remains important for those of refugee background, and may be associated with a worsening pattern of psychological distress. This is consistent with literature that indicates that factors post-settlement such as spending too much time reflecting on past experiences, current international events, separation from family, lack of social support in the host country, marriage stressors and conflict or feeling overwhelmed by resettlement challenges and concerns can contribute to increased levels of psychological distress [29]. It has also been argued that the pattern of distress over time might be explained by the fact that, in the early stages of resettlement, individuals experience a ‘honeymoon’ period of positivity about being in a new country. However, as time progresses, refugees come to face the more sophisticated educational, social and financial challenges of later resettlement [30]. This is particularly relevant if such challenges are not appropriately supported by host communities [30]. In an editorial by Silove and Ekblad [31], it was noted that although preventing trauma experienced by refugees in their homelands is beyond the control of most government officials and clinicians, ensuring that recipient countries provide effective resettlement services is an avenue that has been demonstrated to minimise and mitigate the established links between post-migration stressors and psychological distress. Some of these services which Australia currently provides include encouraging family reunion, offering opportunities for work and education and the provision of targeted specialist mental health services for those in need. However, there is still much work to be done including addressing the unfortunate increase in racism and perceived discrimination, perhaps fuelled by the increase in the threat of global terrorism (past and currents events) [32, 33]. Such experiences of intensified discrimination can only further exacerbate refugee’s feelings of not belonging, fear and insecurity which has been identified as an ongoing mental health hazard with some researchers pointing that receiving countries must increase community awareness about the seriousness of discrimination and its effects [32].

Another factor that may be related to increased levels of psychological distress over time is evidence of underutilisation of mental health services [34] and generalised stigma associated with mental health problems amongst refugees [35]. In addition, when investigating the types of help-seeking amongst refugees, an Australian-based study indicated that Iraqi refugees rarely seek professional mental health services such as a psychiatrist and instead tended to wait until problems become critical or are acute before seeking professional help [36]. Evidence of low levels of mental health literacy in Iraqi refugees, namely, lack recognition of mental health disorders and lack of knowledge of treatment options or professional services reported in an Australian-based study [22] provides an area of potential targeted intervention. One such way forward is to develop appropriate community and individual psycho-education programs for the refugee community, which can have a positive impact on professional help-seeking behaviour [37]. Additionally, developing better guidelines for those working in resettlement agencies, and welfare and educational providers who often assist refugees in early and later stages of migration, so that they are better equipped to respond to mental health crises and offer mental health first aid to affected individuals, would be beneficial. Similarly, General Practitioners and other health professionals should be kept aware that the psychological issues well known to affect those of refugee background can persist for many years, and may even worsen.

Limitations of this study must be noted. These were cross-sectional measures in two disparate groups of refugees, not the same cohort over time. It is possible that the group resettled for longer had worse mental health status when they first arrived, perhaps due to greater stressors pre-migration, and could have in fact improved over time. Another possibility is that those refugees with longer period of resettlement could have been exposed to greater post-displacement traumatic events than those who recently arrived to the country, giving way to poorer mental health. As such, there is a clear need for future research directions to explore psychological distress in Iraqi refugees in a longitudinal cohort, ensuring that it is consistent and replicable by including full demographic profiles, and relevant pre-displacement and post-displacement factors including exposure to potentially traumatic events.

Strengths of this study, in our view, is the relatively large sample size, especially compared to other studies conducted on mental health with refugee populations, with reported sample sizes ranging from 29 to 262 participants within each group [24]. Another strength is that this study focused on refugees from just one country, Iraq, which is different from other studies that have included refugees with different countries of origin [11], heterogeneity that exits amongst different refugees groups with regards to factors such as religion and ethnicity and trauma exposure may impact on results. Although participants in the long-term resettled group were volunteers attending the AMEP classes, by recruiting from colleges in Western Sydney, which has the highest concentration of resettled Iraqi refugees in Australia, some attempt at redressing representativeness was made.

## Conclusions

Overall, our results appear to indicate that those Iraqi refugees with longer periods of resettlement in Australia are experiencing higher levels of psychological distress. Our findings have implications for both for government and non-government funded organisations who should consider the provision of targeted services at critical times beyond the initial arrival period. A method of screening for distress in the immediate to longer resettlement period (post 2 years of arrival) would enable services to provide appropriate mental health interventions to improve well-being and in turn to increase refugee capacity for independence, functionality and productivity.

## Abbreviations

UNHCR: United Nations High Commissioner for Refugees
PTS: Political Terror Scale
PTSD: post-traumatic stress disorder
MDE: major depression episode
NSW RHS: New South Wales Refugee Health Service
AMEP: Adult Migrant English Program
K10: Kessler Psychological Distress Scale
AHS: Australian health survey
